# Impact of systemic inflammatory markers on the response to Hyperthermic IntraVEsical Chemotherapy (HIVEC) in patients with non-muscle-invasive bladder cancer after bacillus Calmette–Guérin failure

**DOI:** 10.1080/2090598X.2021.1874627

**Published:** 2021-01-13

**Authors:** Francesco Chiancone, Marco Fabiano, Maurizio Carrino, Maurizio Fedelini, Clemente Meccariello, Paolo Fedelini

**Affiliations:** Department of Urology, Azienda Ospedaliera di Rilievo Nazionale (A.O.R.N.) Antonio Cardarelli, Naples, Italy

**Keywords:** BCG failure, bladder cancer, chemo-hyperthermia, neutrophil-to-lymphocyte ratio, systemic inflammatory response

## Abstract

**Objectives**: To evaluate the impact of pre- and post-treatment systemic inflammatory markers on the response to Hyperthermic IntraVEsical Chemotherapy (HIVEC) treatment in a cohort of patients with high-grade non-muscle-invasive bladder cancer with bacillus Calmette–Guérin (BCG) failure or intolerance who were unsuitable or unwilling to undergo early radical cystectomy. As a secondary endpoint, we assessed the influence of some demographic, clinical and pathological factors on the response to chemo-hyperthermia.

**Patients and methods**: Between March 2017 and December 2019, 72 consecutive patients were retrospectively analysed. Patients with diseases or conditions that could interfere with systemic inflammatory status or full blood count were excluded. The HIVEC protocol consisted of six weekly intravesical treatments with 40 mg Mitomycin-C diluted in 50 mL distilled water. The drug was heated to a temperature of 43°C. Association of categorical variables with response to HIVEC was evaluated using Yates’ chi-squared test and differences in continuous variable were analysed using the Mann–Whitney test. Logistic regression analysis was performed to define independent predictors of response to HIVEC.

**Results**: Patients who failed HIVEC were more likely to have multiple tumours (*P* = 0.039) at transurethral resection of bladder and a recurrence rate of >1/year (*P* = 0.046). Lower post-HIVEC inflammatory indices [C-reactive protein (*P* = 0.021), erythrocyte sedimentation rate (*P* = 0.027)] and lower pre- (*P* = 0.014) and post-treatment (*P* = 0.004) neutrophil-to-lymphocyte ratio (NLR) values were significantly associated with the response to the HIVEC regimen (no bladder cancer recurrence or progression). In the multivariate analysis, patients with a recurrence rate of >1/year were eight-times more likely to experience failure of HIVEC (*P* = 0.007). Higher pre- (*P* = 0.023) and post-treatment NLR values (*P* = 0.046) were associated with a worse response to the HIVEC regimen.

**Conclusions**: The recurrence rate and systemic inflammatory response markers could be useful tools to predict the likelihood of obtaining a response with the HIVEC regimen. These markers might help to guide patients about the behaviour of the tumour after BCG failure, predicting failure or success of a conservative treatment.

**Abbreviations:** CHT: chemo-hyperthermia; CIS: carcinoma *in situ*; CRP: C-reactive protein; EAU: European Association of Urology; ESR: erythrocyte sedimentation rate; HG: high grade; HIVEC: Hyperthermic IntraVEsical Chemotherapy; ICD: immunogenic cell death; IL: interleukin; MMC: Mitomycin-C; NK: natural killer; NLR: neutrophil-to-lymphocyte ratio; NMIBC: non-muscle-invasive bladder cancer; PLR: platelet-to-lymphocyte ratio; RC: radical cystectomy; SIR: systemic inflammatory response; TURB: transurethral resection of bladder

## Introduction

Intravesical BCG therapy fails in up to 40% of patients with non-muscle-invasive bladder cancer (NMIBC) [1]. Radical cystectomy (RC) represents the ‘gold standard’ in these patients, but carries significant morbidity [1,2]. Delayed RC is associated with a decreased disease-specific survival [3]. Despite this, many bladder preservation strategies have been proposed [4,5]. Hyperthermic IntraVEsical Chemotherapy (HIVEC) is a novel and effective therapeutic choice for patients with BCG-unresponsive NMIBC, showing a median disease-free survival of 17.7 months [6]. HIVEC with Mitomycin-C (MMC) increases the activity of the drug by ~1.4 times. Heated MMC at 43°C has a 10-times higher cytotoxic effect because heat causes instability of the phospholipid bilayer of the cancer cells and increases permeability to MMC [7]. The HIVEC regimen is a safe and efficient alternative also for patients with intermediate- and high-risk NMIBC who have contraindications or cannot tolerate BCG therapy or in cases of critical shortage of BCG. The purpose of the present study was to identify factors that can influence the response to HIVEC in a cohort of patients with NMIBC with BCG failure or intolerance who were unsuitable or unwilling to undergo early RC.

## Patients and methods

### Study design

A total of 72 consecutive patients with NMIBC with BCG failure or intolerance who underwent HIVEC at our institution, between March 2017 and December 2019, were included in this study. The BCG protocol used at our Department consists of a BCG induction (6 weeks) course plus BCG maintenance (3 weeks) courses at 3, 6, 12, 18, 24, 30 and 36 months.

The benefit–risk balance of early and late RC was discussed with patients and written informed consent was obtained. The inclusion criteria were as follows: i) histologically confirmed papillary (Ta or T1) high-grade NMIBC (HG-NMIBC) (WHO 2014) potentially presenting with concomitant carcinoma *in situ* (CIS) detected by transurethral resection of bladder (TURB) after treatment with intravesical BCG and before starting the HIVEC regimen; ii) the criteria of ‘BCG intolerance’ and ‘BCG failure’ according to the European Association of Urology (EAU) Guidelines [1]: BCG refractory [A: T1G3/HG non-muscle-invasive papillary tumour is present at 3 months; B: TaG3/HG non-muscle-invasive papillary tumour or CIS is present at both 3 and 6 months (after a second induction course or the first maintenance course of BCG)], BCG relapsing (recurrence of G3/HG tumour after completion of BCG maintenance, despite an initial response), and BCG intolerance (severe side-effects that prevent further BCG instillation before completing treatment); (iii) patients who completed the HIVEC protocol.

Patients with diseases or conditions that could interfere with systemic inflammatory status or full blood count (e.g. thromboembolism, leukaemia, lymphoma, haematuria, presence of infection, chronic inflammatory diseases, autoimmune diseases, and consumption of steroids) were excluded.

The primary endpoint of the study was to determine the impact of pre- and post-treatment systemic inflammatory markers on the response to HIVEC. As a secondary endpoint, we assessed the influence of some demographic, clinical and pathological factors on the response to chemo-hyperthermia (CHT). Patients underwent a complete routine blood test 1 week before the first hyperthermic instillation and at 1 week after the last one. The neutrophil-to-lymphocyte ratio (NLR) was obtained by dividing the neutrophil count by the lymphocyte count [8]. The platelet-to-lymphocyte ratio (PLR) was obtained by dividing the platelet count by the lymphocyte count.

### Treatment schedule

The induction HIVEC protocol consisted of six weekly intravesical treatments with 40 mg MMC diluted in 50 mL distilled water. The drug was heated to a temperature of 43°C and re-circulated inside the bladder at 200 mL/min for 60 min [7]. All treatments were performed using the Combat BRS system v2.0 (Combat Medical, Wheathampstead, UK).

### Response to HIVEC and follow-up

Response to HIVEC was assessed with urine cytology and cystoscopy performed 6 weeks after the last instillation and subsequent TURB of suspicious lesions. Moreover mapping biopsies (trigone, bladder dome, right, left, anterior and posterior bladder wall) from normal-looking mucosa were performed in all patients [1].

A total of 51 out of 72 patients (70.83%) were classified as a ‘HIVEC responder’ (Group A), while the remaining 21 (29.17%) were classified as ‘HIVEC non-responder’ (Group B). The patients in Group B were defined as all grades of bladder cancer recurrence or progression. Tumour recurrence was reported in 17 patients (five with associated CIS), while progression to muscle-invasive disease was found in four patients. Low-grade recurrent disease was managed with another six weekly HIVEC regimen, while HG recurrences were managed with RC. All patients with HG progression to muscle-invasive disease underwent consequent RC. All Group A patients underwent a maintenance course comprised of three monthly instillations. Subsequently, the disease-free patients underwent another six monthly maintenance course. A CT of the abdomen and pelvis was performed once a year.

### Statistical analysis

All data were collected in a prospectively maintained database and retrospectively analysed. Association of categorical variables with response to HIVEC was evaluated using the Yates’ chi-squared test and differences in continuous variables were analysed using the Mann–Whitney test. Logistic regression model was performed to define independent predictors of response to HIVEC. Nagelkerke *R^2^* was used to understand how much variation in the dependent variable could be explained by the model. Statistical analyses were carried out using the Statistical Package for the Social Sciences (SPSS®), version 23.0 (IBM Corp., Armonk, NY, USA), using a significance level of 0.05.

## Results

### Baseline patient’s characteristics

The baseline patients’ characteristics are summarised in Table 1. The mean (SD) age of the patients was 67.54 (7.96) years. The mean (SD) pre- and post-treatment NLR in all patients was respectively 1.62 (0.45) and 1.39 (0.35).

**Table 1.** Baseline characteristics of the patients who underwent HIVEC treatment

Categorical variables	*n/N* (%)
Sex	
Male	47/72 (65.3)
Female	25/72 (34.7)
Smoking status	
No	30/72 (41.7)
Yes	27/72 (37.5)
Ex-smoker	15/72 (20.8)
Diabetes	13/72 (18.1)
Number of tumours	
Single	21/72 (29.2)
Multiple	51/72 (70.8)
Tumour size, cm	
<3	47/72 (65.3)
≥3	25/72 (34.7)
Recurrence rate	
≤1 year	57/72 (79.2)
>1 year	15/72 (20.8)
Pathological stage	
TaG3	14/72 (19.4)
T1G3	58/72 (80.6)
Concomitant CIS	11/72 (15.3)
Tumour on second TURB	17/72 (23.6)
Prior history of upper tract urothelial carcinoma	7/72 (9.7)
Previously treated with MMC	16/72 (22.2)
BCG failure	
BCG intolerance	13/72 (18.1)
BCG refractory	39/72 (54.2)
BCG relapse	20/72 (2.8)
Continuous variables	Mean (SD)
Age, years	67.54 (7.96)
Body mass index, kg/m^2^	27.35 (4.53)
Pre-HIVEC CRP, mg/L	4.75 (2.58)
Pre-HIVEC ESR, mm/h	7.68 (4.40)
Pre-HIVEC albumin, g/dL	3.77 (0.50)
Pre-HIVEC NLR	1.62 (0.45)
Pre-HIVEC PLR	147.63 (43.60)
Post-HIVEC CRP, mg/L	5.34 (3.93)
Post-HIVEC ESR, mm/h	11.38 (6.71)
Post-HIVEC albumin, g/dL	3.82 (0.53)
Post-HIVEC NLR	1.39 (0.35)
Post-HIVEC PLR	120.30 (27.24)

### Association of clinical and pathological characteristics with response to HIVEC

Patients who failed HIVEC were more likely to have multiple tumours (*P* = 0.039) at TURB and a recurrence rate of >1/year (*P* = 0.046) (Table 2). In terms of blood sample variables, lower post-HIVEC inflammatory indices [C-reactive protein (CRP) (*P* = 0.021), erythrocyte sedimentation rate (ESR) (*P* = 0.027)], lower pre- (*P* = 0.014) and post-treatment NLR (*P* = 0.004) were significantly associated with a good response to HIVEC regimen (Table 2).

**Table 2.** Comparison of categorical and continuous variables between Group A (HIVEC responder) and Group B (HIVEC non-responder)

Categorical Variables, *n/N* (%)	Group A (*n* = 51)	Group B (*n* = 21)		*P*
Sex				
Male	34/51 (66.67)	13/21 (61.90)		0.910
Female	17/51 (33.33)	8/21 (38.10)		0.910
Smoking status				
No	21/51 (41.18)	9/21 (42.86)		0.895
Yes	19/51 (37.25)	8/21 (38.10)		0.841
Ex-smoker	11/51 (21.57)	4/21 (19.05)		0.936
Diabetes	9/51 (17.65)	4/21 (19.05)		0.844
Number of tumours				
Single	19/51 (37.25)	2/21 (9.52)		0.039
Multiple	32/51 (62.75)	19/21 (90.48)		0.039
Tumour size, cm				
<3	37/51 (72.55)	10/21 (47.62)		0.081
≥3	14/51 (27.45)	11/21 (52.38)		0.081
Recurrence rate				
≤1 year	44/51 (86.27)	13/21 (61.90)		0.046
>1 year	7/51 (13.73)	8/21 (38.10)		0.046
Pathological stage				
TaG3	10/51 (19.61)	4/21 (19.05)		0.785
T1G3	41/51 (80.39)	17/21 (80.95)		0.785
Concomitant CIS	7/51 (17.65)	4/21 (9.52)		0.610
Tumour on second TURB	13/51 (13.73)	4/21 (19.05)		0.834
Prior history of upper tract urothelial carcinoma	5/51 (9.80)	2/21 (9.52)		0.688
Previously treated with MMC	14/51 (31.48)	2/21 (9.52)		0.095
BCG failure				
BCG intolerance	11/51 (21.57)	2/21 (9.52)		0.384
BCG refractory	25/51 (49.02)	14/21 (66.67)		0.269
BCG relapse	15/51 (29.41)	5/21 (23.81)		0.847
Continuous variables	Group (*n*)	Mean rank	Z	*P*
Age, years	A (51) B (21)	38.57 31.48	–1.309	0.190
Body mass index, kg/m^2^	A (51) B (21)	34.99 40.17	–0.955	0.339
Pre-HIVEC CRP, mg/L	A (51) B (21)	34.87 40.45	–1.034	0.301
Pre-HIVEC ESR, mm/h	A (51) B (21)	35.30 39.40	–0.766	0.444
Pre-HIVEC albumin, g/dL	A (51) B (21)	35.20 39.67	–0.826	0.414
Pre-HIVEC NLR	A (51) B (21)	32.61 45.95	–2.460	0.014
Pre-HIVEC PLR	A (51) B (21)	34.48 41.40	–1.276	0.202
Post-HIVEC CRP, mg/L	A (51) B (21)	32.87 45.31	–2.300	0.021
Post-HIVEC ESR, mm/h	A (51) B (21)	33.01 44.98	–2.209	0.027
Post-HIVEC albumin, g/dL	A (51) B (21)	35.82 38.14	–0.429	0.673
Post-HIVEC NLR	A (51) B (21)	31.90 47.67	–2.906	0.004
Post-HIVEC PLR	A (51) B (21)	34.62 41.07	–1.189	0.234

### Independent predictive factors for response to HIVEC

The multivariate analysis considered all the factors that at chi-squared and Mann–Whitney testing were significantly associated with HIVEC response in order to better evaluate their role in relation to the primary endpoint (Table 3). The logistic regression model had statistical significance, χ^2^(4) = 28.185, *P* < 0.001. The model explained 46.2% (Nagelkerke *R*^2^) of the variance in HIVEC response and properly classified 80.6% of cases. Patients with a recurrence rate of >1/year were eight-times more likely to experience failure to HIVEC (*P* = 0.007). Higher pre- (*P* = 0.023) and post-treatment NLR values (*P* = 0.046) were associated with a worse response to the HIVEC regimen.

**Table 3.** Logistic regression analysis for assessing the response to the HIVEC regimen

Variables	OR [Exp(B)]	95% CI	*P*
Number of tumours			
Single (reference)	–	–	–
Multiple	1.925	0.382–9.700	0.427
Recurrence rate			
≤1/year (reference)	–	–	–
>1/year	8.002	1.770–36.171	0.007
Pre-HIVEC NLR	4.556	1.236–16.791	0.023
Post-HIVEC CRP	1.127	0.952–1.335	0.163
Post-HIVEC ESR	1.094	0.992–1.206	0.073
Post-HIVEC NLR	3.401	1.024–11.297	0.046

## Discussion

BCG failure is a real challenge in everyday clinical practice because of the high probability of recurrence and progression. RC remains the ‘gold standard’ in these patients [1], even if several salvage therapeutic options have been described as alternatives to RC in the BCG-failure setting [4,5,9,10].

The use of clinical hyperthermia in bladder cancer treatment has a clear rationale. First of all, treatments at temperatures of 41–44°C are cytotoxic to cancer cells, which are unable to tolerate the heat as well as normal cells [11]. Yet, hyperthermia does not increase the toxicity to the patient [12]. Moreover hyperthermia inhibits angiogenesis [13] and increases the activation of natural killer (NK) cells [14].

According to the preliminary results of a randomised (1:1) clinical trial in which 50 patients with high-risk NMIBC were randomised to receive adjuvant BCG or HIVEC, CHT was not inferior to BCG in terms of efficacy as a primary treatment [15,16].

Identification of prognostic factors may help in the selection of specific subgroups of BCG-failure patients who could benefit from early RC. In our retrospective study, the number of tumours, prior recurrence rate, post-HIVEC inflammatory indices (CRP and ESR), pre- and post-treatment NLR may help to identify patients that may fail the HIVEC induction course.

Multivariate analysis showed that the recurrence rate and pre-treatment NLR values were independent factors of failure at first follow-up. Moreover, higher post-HIVEC NLR values could be considered a biomarker for poor response to CHT.

According to the EAU Guidelines [1], concomitant CIS, multiple, large and recurrent tumours are already considered characteristics of highest risk. Moreover, analysing a group of 1812 patients with intermediate- and high-risk NMIBC treated with BCG, the prior disease recurrence rate and numbers of tumours were the most important prognostic factors determining tumour recurrence [17].

Furthermore, the systemic inflammatory response (SIR) could provide some additional information about the possible response to HIVEC. Few published studies have investigated the relationship between SIR markers and NMIBC. The NLR before BCG treatment was associated with both survival and oncological outcomes in patients with NMIBC [18].

Ferro et al. [19] demonstrated that NLR was a predictor of residual HG disease at re-TURB at univariate but not at multivariate analysis. Racioppi et al. [20] reported that higher preoperative NLR could be predictive of poor BCG response in multivariate analysis.

Several studies have evaluated the association between NLR and post-RC survival outcomes [21]. Interestingly, Kang et al. [22] showed that post-treatment NLR measured in the early post-RC period was an independent factor of poor oncological prognosis.

Some chemotherapeutic drugs (such as anthracyclines and oxyplatin) induce immunogenic cell death (ICD), resulting in increased immunity. However, many chemotherapeutic agents, including MMC, etoposide and cisplatin, do not cause ICD [23]. It is possible that CHT induces ICD or activates the immune system through heat shock proteins or other factors. Hyperthermia has an important impact on the immune system resulting in augmented activation of NK cells [13] that destroy heat-stressed cancer cells. Moreover, thermotherapy induces heat shock proteins expression on the cancer cell surface. As a consequence, cancer cells actively participate in their programmed death through the natural process of apoptosis [24].

The SIR activated by cancer induces a pro-tumour inflammatory state, facilitating tumour growth, recurrence, and progression. In particular, neutrophils and lymphocytes have an inhibitory and promoting action, respectively, on the immune system and they can reflect the inflammatory and immune response of the patients.

Inflammatory responses induce neutrophilia, lymphocytopenia and high production of pro-angiogenic, anti-apoptotic and growth factors, that can stimulate tumour growth, angiogenesis, and metastasis [25]. Moreover, peripheral neutrophils can inhibit the cytotoxic T lymphocytes and NK cells [26]. In addition, the increase in tumour-infiltrating lymphocytes has been associated with improved prognosis in various tumours [27].

Consequently, a high NLR value could indicate both an increased neutrophil-dependent inflammatory response and a diminished lymphocyte-dependent immune response [28], and might represent an interesting biomarker of the host–tumour interactions.

CRP is a non-specific marker of inflammation. It is considered an important prognostic biomarker in various malignancies including kidney and urothelial tumours.

Some cancer cells express CRP and secrete interleukin 6 (IL-6) and IL-8, which stimulate CRP synthesis in the liver. Moreover, CRP positivity creates a favourable microenvironment for the tumour cells through acute inflammatory cytokine network system maintenance [29].

The inflammatory state appears to increase the irritative and voiding symptoms during and after CHT. Bladder instillations with dexamethasone and sodium hyaluronate can control the local inflammatory state. Moreover, the oral administration of nuciferine may inhibit the production of lipopolysaccharide-induced inflammatory cytokine, IL-6 and TNF-α, with good control of irritative symptoms [30].

The small cohort of patients and the retrospective nature are some limitations of the present study. Moreover, the present study is limited by its short follow-up period; this may have resulted in some patients being falsely deemed as ‘responders’ due to the short follow-up alone. Another limitation is that albumin, CRP, ESR, NLR and PLR do not characterise the whole SIR state. Nevertheless, we believe that the present study provides new evidence showing a possible association between SIR and risk of failure of HIVEC.

In conclusion, the recurrence rate and pre-treatment NLR could be useful tools to predict the likelihood of obtaining a response to the HIVEC regimen. Moreover, post-treatment NLR can be considered a biomarker for response to the induction course of CHT. These markers might help to guide patients about the behaviour of the tumour after BCG failure, giving a high probability of failure or success of a conservative treatment. Additional larger scale prospective trials are needed to validate these results.

## References

[1] Babjuk M, Böhle A, Burger M, et al. EAU guidelines on non-muscle-invasive urothelial carcinoma of the bladder: update 2016. Eur Urol. 2017;71:447–461. 27324428 10.1016/j.eururo.2016.05.041

[2] Bada M, De Concilio B, Crocetto F, et al. Laparoscopic radical cystectomy with extracorporeal urinary diversion: an Italian single-center experience with 10-year outcomes. Minerva Urol Nefrol. 2020;72:641–643. 32550634 10.23736/S0393-2249.20.03850-3

[3] Raj GV, Herr H, Serio AM, et al. Treatment paradigm shift may improve survival of patients with high risk superficial bladder cancer. J Urol. 2007;177:1283–1286. 17382713 10.1016/j.juro.2006.11.090

[4] Sari Motlagh R, Pradere B, Mori K, et al. Bladder-preserving strategies for Bacillus Calmette-Guérin unresponsive non-muscle invasive bladder cancer; where are we and what will be expected? Curr Opin Urol. 2020;30:584–593. 32304379 10.1097/MOU.0000000000000792

[5] Li R, Sundi D, Zhang J, et al. Systematic review of the therapeutic efficacy of bladder-preserving treatments for non-muscle-invasive bladder cancer following intravesical Bacillus Calmette-Guérin. Eur Urol. 2020;78:387–399. 32143924 10.1016/j.eururo.2020.02.012 PMC7771323

[6] de Jong JJ, Hendricksen K, Rosier M, et al. Hyperthermic intravesical chemotherapy for BCG unresponsive non-muscle invasive bladder cancer patients. Bladder Cancer. 2018;4:395–401. 30417050 10.3233/BLC-180191 PMC6218110

[7] Chiancone F, Carrino M, Fedelini M, et al. Intravesical thermo-chemotherapy using the combined antineoplastic thermotherapy bladder recirculation system for BCG-failure patients: a single center experience. Eur Urol Suppl. 2019;18:e3248.

[8] Forget P, Khalifa C, Defour JP, et al. What is the normal value of the neutrophil-to-lymphocyte ratio? BMC Res Notes. 2017;10:12. 28057051 10.1186/s13104-016-2335-5 PMC5217256

[9] Mansour AM. Bacille Calmette-Guérin dosage; challenging dogmas and changing paradigms. Arab J Urol. 2015;13:244. 26609441 10.1016/j.aju.2015.09.006 PMC4656808

[10] Li R, Spiess PE, Kamat AM. Treatment options for patients with recurrent tumors after BCG therapy: are we ignoring the obvious? Eur Urol. 2018;74:405–408. 29685644 10.1016/j.eururo.2018.04.012

[11] Matsuoka Y, Taoka R, Xia Z, et al. Hyperthermic therapy using warm sterile water enhances cytocidal effects on bladder cancer cells. Scand J Urol. 2020;54:65–69. 31928301 10.1080/21681805.2019.1708967

[12] Roca C, Primo L, Valdembri D, et al. Hyperthermia inhibits angiogenesis by a plasminogen activator inhibitor 1-dependent mechanism. Cancer Res. 2003;63:1500–1507. 12670896

[13] Sawaji Y, Sato T, Takeuchi A, et al. Anti-angiogenic action of hyperthermia by suppressing gene expression and production of tumour-derived vascular endothelial growth factor in vivo and in vitro. Br J Cancer. 2002;86:1597–1603. 12085210 10.1038/sj.bjc.6600268 PMC2746582

[14] Dayanc BE, Beachy SH, Ostberg JR, et al. Dissecting the role of hyperthermia in natural killer cell mediated anti-tumor responses. Int J Hyperthermia. 2008;24:41–56. 18214768 10.1080/02656730701858297

[15] Sousa A, Piñeiro I, Rodríguez S, et al. Recirculant hyperthermic IntraVEsical chemotherapy (HIVEC) in intermediate-high-risk non-muscle-invasive bladder cancer. Int J Hyperthermia. 2016;32:374–380. 26915466 10.3109/02656736.2016.1142618

[16] González Padilla DA, González Diaz A, Miranda-Utrera N, et al. HIVEC HR:Chemohyperthermia with mitomycin C vs BCG for high-risk non-muscle invasive bladder cancer. Preliminary results from a randomized controlled trial. Eur Urol Suppl. 2019;18:e768–e770.

[17] Cambier S, Sylvester RJ, Collette L, et al. EORTC nomograms and risk groups for predicting recurrence, progression, and disease-specific and overall survival in non-muscle-invasive Stage Ta-T1 urothelial bladder cancer patients treated with 1–3 years of maintenance Bacillus Calmette-Guérin. Eur Urol. 2016;69:60–69. 26210894 10.1016/j.eururo.2015.06.045

[18] Yuk HD, Jeong CW, Kwak C, et al. Elevated neutrophil to lymphocyte ratio predicts poor prognosis in non-muscle invasive bladder cancer patients: initial intravesical Bacillus Calmette-Guerin treatment after transurethral resection of bladder tumor setting. Front Oncol. 2019;8:642. 30705874 10.3389/fonc.2018.00642 PMC6344445

[19] Ferro M, Di Lorenzo G, Buonerba C, et al. Predictors of residual T1 high grade on re-transurethral resection in a large multi-institutional cohort of patients with primary T1 high-grade/Grade 3 bladder cancer. J Cancer. 2018;9:4250–4254. 30519326 10.7150/jca.26129 PMC6277616

[20] Racioppi M, Di Gianfrancesco L, Ragonese M, et al. Can neutrophil-to-lymphocyte ratio predict the response to BCG in high-risk non muscle invasive bladder cancer? Int Braz J Urol. 2019;45:315–324. 30785697 10.1590/S1677-5538.IBJU.2018.0249 PMC6541147

[21] Gondo T, Nakashima J, Ohno Y, et al. Prognostic value of neutrophil-to-lymphocyte ratio and establishment of novel preoperative risk stratification model in bladder cancer patients treated with radical cystectomy. Urology. 2012;79:1085–1091. 22446338 10.1016/j.urology.2011.11.070

[22] Kang M, Jeong CW, Kwak C, et al. The prognostic significance of the early postoperative neutrophil-to-lymphocyte ratio in patients with urothelial carcinoma of the bladder undergoing radical cystectomy. Ann Surg Oncol. 2016;23:335–342. 26152275 10.1245/s10434-015-4708-8

[23] Gebremeskel S, Johnston B. Concepts and mechanisms underlying chemotherapy induced immunogenic cell death: impact on clinical studies and considerations for combined therapies. Oncotarget. 2015;6:41600–41619. 26486085 10.18632/oncotarget.6113 PMC4747176

[24] Ahmed K, Tabuchi Y, Kondo T. Hyperthermia: an effective strategy to induce apoptosis in cancer cells. Apoptosis. 2015;20:1411–1419. 26354715 10.1007/s10495-015-1168-3

[25] Mantovani A, Allavena P, Sica A, et al. Cancer-related inflammation. Nature. 2008;454:436–444. 18650914 10.1038/nature07205

[26] Petrie HT, Klassen LW, Kay HD. Inhibition of human cytotoxic T lymphocyte activity in vitro by autologous peripheral blood granulocytes. J Immunol. 1985;134:230–234. 3871101

[27] Fu Q, Chen N, Ge C, et al. Prognostic value of tumor-infiltrating lymphocytes in melanoma: a systematic review and meta-analysis. Oncoimmunology. 2019;8:1593806. 31143514 10.1080/2162402X.2019.1593806 PMC6527267

[28] Ku JH, Kang M, Kim HS, et al. The prognostic value of pretreatment of systemic inflammatory responses in patients with urothelial carcinoma undergoing radical cystectomy. Br J Cancer. 2015;112:461–467. 25584490 10.1038/bjc.2014.631 PMC4453653

[29] Ohno Y. Role of systemic inflammatory response markers in urological malignancy. Int J Urol. 2019;26:31–47. 30253448 10.1111/iju.13801

[30] Chiancone F, Carrino M, Fedelini M, et al. The role of Protopine associated with Nuciferine in controlling adverse events during hyperthermic intravesical chemotherapy instillations. A nutraceutical approach to control adverse event during intravesical instillations. Arch Ital Urol Androl. 2020;92:177. 10.4081/aiua.2020.3.177 33016036

